# The Relationship between Selected CNR1, MC4R, LEP, FTO and VDR Gene Polymorphisms and Several Basic Toxicological Parameters Among Persons Occupationally Exposed to Arsenic, Cadmium and Lead

**DOI:** 10.3390/jcm9041040

**Published:** 2020-04-07

**Authors:** Tomasz Matys, Anna Szymańska-Chabowska, Katarzyna Bogunia-Kubik, Beata Smyk, Małgorzata Kamińska, Grzegorz Mazur, Rafał Poręba, Paweł Gać

**Affiliations:** 1 Department of Internal Medicine, Occupational Diseases, Hypertension and Clinical Oncology, Wroclaw Medical University, Borowska 213, PL 50-556 Wroclaw, Poland; t.matys@interia.pl (T.M.); aszyman@mp.pl (A.S.-C.); beata.smyk@umed.wroc.pl (B.S.); grzegorz.mazur@umed.wroc.pl (G.M.); rafal.poreba@umed.wroc.pl (R.P.); 2 Laboratory of Clinical Immunogenetics and Pharmacogenetics, Hirszfeld Institute of Immunology and Experimental Therapy, Polish Academy of Sciences, Weigla 12, PL 53-114 Wroclaw, Poland; katarzyna.bogunia-kubik@hirszfeld.pl (K.B.-K.); malgorzata.kaminska@hirszfeld.pl (M.K.); 3 Department of Hygiene, Wroclaw Medical University, Mikulicza-Radeckiego 7, PL 50-368 Wroclaw, Poland

**Keywords:** arsenic, cadmium, lead, single nucleotide polymorphism, zinc protoporphyrin

## Abstract

The purpose of this work was to assess the influence of selected CNR1, MC4R, LEP, FTO and VDR FOKI gene polymorphisms on blood and urine concentration markers of lead, cadmium and arsenic in a population directly exposed to these metals. Eighty-five people exposed to lead, arsenic and cadmium were qualified to take part in the study. Standard urine samples and 25 mL of venous blood from each worker were collected to assay basic laboratory and toxicological markers as well as selected single nucleotide polymorphisms (SNPs) within CNR1—cannabinoid receptor 1 gene (*rs806368, rs806381, rs1049353, rs12720071*), MC4R—melanocortin 4 receptor gene (*rs17782313*), LEP—leptin promoter gene (*rs7799039*), FTO—alpha-ketoglutarate-dependent dioxygenase gene (*rs9939609*) and VDR—vitamin D receptor (*rs10735810*) genes. It appeared that, except for the MC4R SNP, all the other polymorphisms were found to be associated with various laboratory parameters. Arsenic concentration in urine was associated with all four CNR1 and LEP SNPs, while cadmium concentration in blood was affected by the VDR polymorphism. Moreover, some significant relationships were also observed between CNR1 *rs1049353* and FTO *rs9939609* gene variants and markers of lead exposure. These results imply SNPs within genes coding for proteins involved in development of metabolic syndrome may be of prognostic value for persons directly exposed to lead, cadmium and arsenic.

## 1. Introduction

Metabolic syndrome is an important global clinical problem and a challenge for modern medicine [1]. Metabolic syndrome was firstly described in specified detail in 1988 by Reaven. He suggested that it has four components: visceral obesity, hyperglycemia, arterial hypertension and dyslipidemia, described as hyper triglycerides, low HDL cholesterol fraction and high non-HDL fraction [2,3].

Research that aims to explain the causes of metabolic syndrome and its epidemic occurrence is based, on the one hand, on genetic and genotypic analyses and, on the other hand, observation of influence of the external environment [4,5].

Metabolic syndrome is multi-genetic. Among the genes with the strongest relationship with metabolic syndrome, CNR1—cannabinoid receptor 1 gene, LEP—leptin promoter gene, FTO—alpha-ketoglutarate-dependent dioxygenase gene, MC4R—melanocortin 4 receptor gene and VDR—vitamin D receptor stand out.

CNR1 (cannabinoid receptor 1) is a G protein-coupled receptor activated by endogenous and exogenous cannabinoids. They are found mostly in the central nervous system, in the cerebellum, nucleus accumbens and several other brain regions responsible for hunger, satiety and the reward system [6]. Several specific single nucleotide polymorphisms of the CNR1 gene (located in 6q15) were found (*rs806381*, *rs806368*, *rs1049353*, *rs12720071*), as well as several possible combinations. The most popular, A to G transition in *rs1049353,* leads to higher body mass index and wider waist circumference [7]. Another studied polymorphism was leptin (LEP), a hormone made mostly by adipocyte cells. It inhibits hunger and stimulates the sympathetic nervous system. Despite its seemingly straightforward effect on the human organism, current research suggests that leptin polymorphism is not a relevant obesity marker [8]. Next, MC4R polymorphism was studied. Melanocortin 4 receptor is a known genetic obesity marker [9]. Yet, the mechanism remains unclear, and homozygous CC (*rs17782313*) tends to be associated with higher body mass index and insulin resistance [10]. Another typical obesity polymorphism is the FTO gene responsible for fat mass and an obesity-associated protein also known as alpha-ketoglutarate-dependent dioxygenase. Certain variants of this enzyme, active mostly in the central nervous system, are also correlated with higher BMI and obesity [11]. Finally, VDR (vitamin D receptor) FokI polymorphism was studied. This particular receptor is responsible for most of the comprehensive activity of vitamin D. Some variants are known to be responsible for different bone density, some are correlated with renin activity and some could even directly induce obesity [12].

According to current knowledge, exposure to metals may affect the development of metabolic syndrome. There are more and more reports confirming the existence of such a relationship.

Using data from the 2011–2014 National Health and Nutrition Examination Survey, Bulka et al. evaluated associations between essential and toxic metals exposure and metabolic syndrome [13]. The positive correlations observed for arsenic exposure were due to an elevated prevalence of high blood pressure, low HDL cholesterol and high triglycerides among people with greater exposures. On the other hand, greater lead and cadmium co-exposures were related to a lower prevalence of dyslipidemia and abdominal obesity.

On the contrary, an analysis based on the Korea National Health and Nutrition Examination Survey (KNHANES) found that a higher prevalence of metabolic syndrome was associated with higher blood lead levels in the Korean population [14].

In a study by Wang et al., blood and urinary markers of 18 heavy metals among 9537 adults in NHANES 2003–2014 were evaluated. This study suggests that cumulative exposure to heavy metals as mixtures is associated with obesity and its related to chronic conditions such as hypertension and diabetes type II [15].

Luzhetskyi and co-authors proved that children with higher serum levels of cadmium and arsenic (1.4–2.0 times vs. the reference group) demonstrated 2.2 times more frequent endocrine diseases, up to 2.7 times more frequent obesity-related diseases, when compared to the reference group. Metabolic disorders in that group were associated with some lipid metabolism changes [16].

Similarly, study of Kawakami et al. demonstrated that exposure to cadmium caused a reduction of adipocyte size and the modulation of adipokine expression. The reduction in adipocyte size by Cd may arise from an imbalance between lipid synthesis and lipolysis. In addition, the expression levels of leptin, adiponectin and resistin decreased in adipocytes. So, exposure to Cd may induce unusually small adipocytes and modulate the expression of adipokines differently from the case of physiologically small adipocytes, and it may accelerate the risk of developing insulin resistance and type 2 diabetes [17].

Numerous data concerning potential arsenic, cadmium and lead effects on development of obesity and metabolic syndrome have inspired us to pose a hypothesis that concentrations of these metals in people occupationally exposed are related to selected gene polymorphisms and determine metabolic disorders.

The purpose of this work was to assess the influence of selected single nucleotide polymorphisms (SNPs) coding for proteins involved in development of metabolic syndrome with the previously mentioned selected gene polymorphisms on blood and urine concentration markers of lead, cadmium and arsenic in a population directly exposed to these metals.

## 2. Materials and Methods

Eighty-five people, employees of a copper smelter and refinery, were qualified to be in the study. Inclusion criteria for the study were employment in workplaces exposed to arsenic, cadmium and lead (metal concentrations >0.1 maximum admissible concentration (MAC)) and work-related exposure to metals for at least 0.25 years. Size and quality of environmental exposure for all subjects included in the study were similar because all subjects resided in the same region. Only people who lived in the region for a long time (for at least 3 years) were included in the study group. The data and biological sample were collected in June 2016. There were 67 men and 18 women, and age ranged from 26 to 67 years. Thirty-three participants were obese, 34 of them were previously diagnosed with hypertension and 37 of them were smokers. Full clinical characteristics of the group are shown in Table 1, and basic laboratory characteristics of the population are shown in Table 2.

All the participants were asked to fill in a questionnaire about their medical history and lifestyle. Next, basic anthropometric measurements were taken. We also took standard urine samples and 25 mL of venous blood from each worker just after finishing their work shift to assay basic laboratory and toxicology markers as well as selected single nucleotide polymorphisms.

Blood count, fasting glucose, HbA1C, lipids (total cholesterol, HDL and LDL cholesterol, triglycerides) and calcium-phosphate balance markers (calcium, phosphorus, 25-OH-D_3_ and parathormone) were determined by standard methods in accordance with the manufacturer’s instructions.

We also determined concentrations of lead (Pb-B), cadmium (Cd-B), zinc protoporphyrins in blood (ZnPP) and arsenic in urine (As-U). Blood lead and cadmium concentrations were measured by graphite furnace atomic absorption spectrometry Solaar M6 (Thermo Elemental, UK). The calibration curves of lead and cadmium were prepared with blood standards of certified reference material (BCR IRMM). Both methods are routinely monitored by determination of reference material (Recipe) and participation in an intercomparison program for toxicological analyses in biological materials, G-EQUAS. The measurement was calculated as micrograms per liter (µg/L), and the biological exposure limits were 500 µg/L for lead and 5 µg/L for cadmium according to recommendations of the National Hygiene Institute and Institute of Occupational Health. ZnPP was measured using a rapid fluorometric screening method by means of Hematofluorymeter ProtoFluor (Helena Laboratories, Beaumont, Texas, USA). Total urine arsenic concentration was measured by Hydride Generation Atomic Absorption Spectrometry (HGAAS) using the VP100 Continuous Flow Vapour System. To determine the calibration curve, the reference material ClinCal^®^ Urine Calibrator (Recipe) was used. We monitored the accuracy of the analytical method by analyzing samples of a reference material, Seronorm Trace Elements Urine (SERO AS, Oslo Norway), and participation in an intercomparison program for toxicological analyses in biological materials, G-EQUAS. The biological exposure limit proposed by the National Hygiene Institute and Institute of Occupational Health for arsenic in urine is 35 µg/L (35 µg/g creatinine).

In the study we analyzed selected single nucleotide polymorphisms (SNPs) for cannabinoid receptor 1 gene (CNR1), melanocortin 4 receptor gene (MC4R), leptin (LEP), alpha-ketoglutarate-dependent dioxygenase gene (FTO) and vitamin D receptor FokI. In the present study, the following eight SNPs were selected: *rs17782313* (T>C), located upstream of the MC4R gene on chromosome 18q22; *rs7799039* (G>A), a common promoter polymorphic site within the LEP gene on chromosome 7q31.3; *rs9939609* (G>A), a SNP within intron 1 of the FTO gene on chromosome 16q12.2; *rs10735810* (FokI) (C>T), located within exon 2 of the VDR gene on chromosome 12q11; and four polymorphisms of the CNR1 gene located on chromosome 6q15 (one intronic SNP *rs806381* A>G, *rs806368* T>C within 3′UTR, *rs1049353* a synonymous G>A polymorphism (within the splicing site), and *rs12720071* A>G, a substitution in 3′UTR). Genotyping was performed by real-time polymerase chain reaction (PCR) amplifications and a melting curve analysis using a LightSNiP typing assay (TIB-MolBiol, Berlin, Germany). Real-time PCR was carried out on a LightCycler 480 Real-Time PCR system (Roche Diagnostics, Rotkreuz, Switzerland) in accordance with the conditions recommended by the manufacturers.

The research was compliant with Good Clinical Practice guidelines, The Declaration of Helsinki and was based on consent from a local Bioethical Committee (No KB-398/2018, date: 25.06.2018).

Statistical analyses were calculated using the statistical program STATISTICA 13 (Dell Inc., Tulsa, Oklahoma, USA). For the quantitative variables, the arithmetic mean (X), the median value (Me), the standard deviation (SD) as well as the minimal (Min) and maximal (Max) values of assayed parameters were calculated in the studied groups. Distribution of variables was tested using the Lilliefors and W-Shapiro–Wilk tests. In the case of independent, quantitative variables having normal distributions, a t test for independent variables and the analysis of variances ANOVA (unifactorial parametric) were used in the further statistical analysis. The U test of Mann–Whitney or a non-parametric equivalent of Kruskal–Wallis ANOVA analysis of variance test were used in the case of variables with non-normal distributions. The significant differences between the arithmetic means were estimated using a post-hoc Newman–Keuls test. Results for the nominal variables were presented in percentages. In order to assess the relations between studied variables, a correlation analysis was performed. In the case of variables having a normal distribution, Pearson’s r was calculated, and for the variables with a distribution other than normal, the Spearman’s r correlation coefficient was used. Results at the level of *p* < 0.05 were regarded as statistically significant.

## 3. Results

The mean blood calcium, phosphorus, 25-hydroxyvitamin D and parathormone concentrations in the occupationally exposed group were accordingly 9.69 ± 0.33 mg/dL, 3.43 ± 0.66 mg/dL, 20.81 ± 7.69 µg/L and 46.68 ± 20.02 ng/L. These are presented in Table 2.

Arsenic concentration was 11.74 ± 9.37 µg/L, cadmium was 0.84 ± 0.80 µg/L, lead was 199.23 ± 117.02 µg/L and ZnPP was 47.94 ± 30.64 μg/dL. A total of 3.5% of employees had a urine arsenic concentration higher than the norm of the allowable concentration in biological material (determined as a maximum of 35 μg/L). Totals of 1.2% and 16.5% of workers, respectively, had a blood lead concentration and blood zinc protoporphyrin concentration higher than the norms of the allowable concentration in biological material (determined as maximums of 500 and 70 μg/L, respectively). Nobody in the study group was characterized by exceeding the norm of the permissible blood cadmium concentration. These are presented in Table 3.

Distributions of alleles and genotypes of selected SNPs in the studied population are shown in Table 4. Most genotypes had frequencies exceeding 10%. The CRN1 *rs12720071* GG homozygosity was the rarest and was detected in one individual only (1.2%). The other rare genotypes were MC4R *rs17782313* GG and CNR1 *rs1049353* AA homozygosity observed both in five (5.9%) cases, and the CNR1 *rs806386* CC genotype was found in seven (8.2%) cases. These distributions closely resemble those described for other European populations.

The results of comparative analyses of arsenic urine concentrations of subgroups based on genotype criteria and alleles of single nucleotide polymorphisms of genes CNR1, MC4R, LEP, FTO and VDR FokI are presented in Table 5. We have proved that homozygosity AA in locus *rs806381* in the CNR1 gene is related to a statistically significant lower arsenic concentration, compared to heterozygosity AG and homozygosity GG, and the presence of allele G in this locus is associated with a significantly higher arsenic urine concentration. In locus *rs12720071* of the CNR1 gene, homozygotes GG have statistically significant higher arsenic concentrations than heterozygotes AG and homozygotes AA, and allele A in this locus can be associated with a statistically significant lower arsenic concentration. The analysis also has shown that allele A in locus *rs1049353* of the CNR1 gene can be responsible for lower arsenic concentrations, and allele G in locus *rs7799039* of gene LEP is responsible for significantly higher arsenic concentrations in the studied population.

The results of comparative analyses of cadmium blood concentrations of subgroups based on genotype criteria and alleles of single nucleotide polymorphisms of genes CNR1, MC4R, LEP, FTO and VDR FokI are presented in Table 6. We have proved that allele T in locus *rs10735810* of VDR FokI gene can be a factor responsible for the significantly lower cadmium concentration in the studied population.

Similarly, the results of comparative analyses of lead blood concentrations of subgroups based on genotype criteria and alleles of single nucleotide polymorphisms of genes CNR1, MC4R, LEP, FTO and VDR FokI are presented in Table 7. In the studied population, homozygotes GG in locus *rs1049353* of the CNR1 gene have a significantly higher blood lead concentration compared to heterozygotes AG and homozygotes AA. The presence of allele A in the locus is correlated with a statistically relevant lower lead blood concentration.

The results of comparative analyses of zinc protoporphyrin blood concentrations of subgroups based on genotype criteria and alleles of single nucleotide polymorphisms of genes CNR1, MC4R, LEP, FTO and VDR FokI are presented in Table 8. It was proven that heterozygosity AG in locus *rs1049353* of the CNR1 gene may result in a statistically lower ZnPP concentration compared to homozygosity AA and GG, and allele A is responsible for lower ZnPP concentrations. Apart from that, we documented that allele A in locus *rs9939609* of the FTO gene is responsible for higher ZnPP concentration.

In the correlation analysis we found statistically significant, linear correlations between cadmium concentration and white blood cell count (r = 0.22, *p* = 0.040), ZnPP concentration and platelets (r = 0.25, *p* = 0.020), ZnPP concentration and phosphorus in blood (r = 0.23, *p* = 0.035) as well as ZnPP and vitamin D (r= −0.22, *p* = 0.032).

In the last part of the study, multivariate regression analysis was performed, and the following significant models were observed:

As-U = 1.330 allele G in *rs806381* gene CNR1 − 4.274 allele A in *rs1049353* gene CNR1 − 18.415 allele A in *rs12720071* gene CNR1 + 2.291 allele G in *rs7799039* gene LEP + 0.424 BMI + 0.160 age + 5.324 diabetes ± 4.877.

Cd-B = − 0.439 allele T in *rs10735810* gene VDR FokI + 0.814 smoking − 0.023 HDL cholesterol + 0.014 BMI ± 0.709.

Pb-B = − 77.411 allele A in *rs1049353* gene CNR1 + 62.804 hypertension + 82.478 diabetes − 44.553 phosphorus ± 0.709.

Based on the obtained regression models it was shown that allele G in *rs806381* gene CNR1, allele G in *rs7799039* gene LEP, higher BMI, older age and diabetes were independently associated with higher As-U concentration; while allele A in *rs1049353* gene CNR1 and allele A in *rs12720071* gene CNR1 were independently associated with lower As-U concentration. It was shown that allele T in *rs10735810* gene VDR FokI and higher HDL cholesterol concentration were independently associated with lower Cd-B concentration, while smoking and higher BMI were independently associated with higher Cd-B concentration. Finally, allele A in *rs1049353* gene CNR1 and higher phosphorus concentration were independently associated with a lower Pb-B concentration, while hypertension and diabetes were independently associated with a higher Pb-B concentration (Table 9).

## 4. Discussion

The relationship between some metals and body mass, obesity development and central hunger regulation is widely discussed and confirmed in numerous studies. Many studies focus on genetic and epigenetic mechanisms of obesity evolution. In the study of Tyrrell et al., Pb-exposed animals showed elevated hepatic triglyceride levels and increased expression of the gluconeogenic genes PEPCK and glucose-6-phosphatase [18]. In cultured rat hepatoma cells, treatment with Pb stimulated PEPCK and glucose-6-phosphatase gene expression, suggesting a possible direct effect of Pb on hepatic gluconeogenic gene expression. Vidal et al. proved that elevated maternal blood Cd levels were associated with lower birth weight, and higher maternal blood Cd levels were also associated with lower methylation at the PEG3 or at the MEG3 in methylated regions of newborn DNA [19].

Our study is either the first or one of the very few studies trying to determine the relationship between single nucleotide polymorphisms of genes involved in development of metabolic syndrome and toxicological parameters. Although the literature is poor, we decided to proceed with selected genes as they are the best studied in the aspect of metabolic syndrome. We found several statistically important correlations. However, we were unable to compare our results with other studies, as there have been none. On the other hand, there are numerous studies concerning studied polymorphisms and various other parameters. A comprehensive discussion of the subject will extend the framework of this research. Therefore, we decided to discuss a few examples to show the scientific and clinical importance of our study, as well as the need to continue the research.

Until now, we were unable to find other studies showing correlations between CNR1 polymorphism locus *rs1049353* and arsenic concentration. Moreover, data concerning this polymorphism and its correlation with BMI parameters are inconclusive and often contradictory [7]. In our research we were able to determine a significant correlation: allele G is correlated with higher arsenic concentration. This poses the following question: what is the reason for such correlation? Do people with allele G consume more food, as some studies show [20], therefore absorbing more arsenic and resulting in a higher arsenic concentration? Or, on the other hand, does a greater amount of fat tissue enable a higher arsenic concentration? Whatever the mechanism could be, we have found a genetic predisposition to higher arsenic concentration. This requires further study, especially in light of the growing obesity epidemic.

In our study we discovered that allele G in locus *rs7799039* of gene LEP is correlated with a markedly higher arsenic concentration. The authors were not able to find any other research to support or undermine our result. Nevertheless, the leptin polymorphism has been heavily studied recently. Carriers of A allele in the studied locus tend to have lower LDL and total cholesterol [21]. Another recent study shows association between leptin polymorphism and coronary artery disease and hypertension [22]. This finally leads us to the question about the role of arsenic in development of metabolic syndrome and cardiovascular diseases. This role is yet to be determined.

We were also able to find that allele T in locus *rs10735810* of VDR FokI gene is associated with lower cadmium concentration. Again, it seems that it is the first attempt to assess this correlation. There are single studies on metals and VDR FokI polymorphism. In the study by Szymanska-Chabowska et al., they were able to determine an association between another locus of VDR FokI gene (*rs2228570*) and concentration of lead and ZnPP [23]. Our study provides more input on the matter, especially that some research suggests there is no simple connection between cadmium concentration and vitamin D concentration [24].

In studied population, we were able to determine a significant correlation between the CNR1 polymorphism and lead and ZnPP concentrations. Again, this is the first research proving this relation. However, polymorphism of CNR1 is being thoroughly studied. It was found that *rs1049353* polymorphism was associated with specific changes in brain morphology as well as with evolution of positive symptoms in schizophrenia [25]. This poses a question about the role of lead in the development of psychiatric disorders and brain reconstruction.

As for the linear correlation between cadmium concentration and white blood cell count, our research is compliant with many previous studies, including the most recent ones [26,27]. Correlations of ZnPP concentration and platelets, ZnPP concentration and phosphorus in blood as well as ZnPP and vitamin D were not found in previous studies [23].

Finally, it should be mentioned that, when analyzing multiple polymorphisms affecting a single variable (e.g., blood concentration of a particular metal), it is necessary to consider the possible compensatory effects of polymorphisms. In the current study, this compensation in individuals may be due to the effect of polymorphisms on urinary arsenic levels. If a person is a carrier of allele G in loci *rs806381* CNR1 gene and *rs7799039* LEP gene, and at the same time a carrier of allele A in loci *rs1049353* and *rs12720071* CNR1 gene, the increase in As-U concentration due to the impact of the first two alleles may be reduced as a result of the impact of the second two alleles.

The authors of this work see two main limitations of this study. First, there is little or no research to compare and confront our data. Our study was conducted with the highest standards; we found statistically significant correlations, but still, further research needs to be conducted to confirm our results. Secondly, our study is based on a relatively small population (85 persons) for genetic study. This results in poor representation of certain alleles (allele G in locus *rs12720071* CNR1—18.8%; allele C in locus *rs17782313* MC4R—37.6%). As for the first allele, we were able to obtain statistically significant correlation. If statistically important correlations are found in a relatively small group, the correlation is strong and it will be even more visible in larger groups. Finally, showing specific correlations between polymorphisms and metal concentrations is just the first step in understanding their complex role in the human organism; yet, it is an important step forward.

Relationships that we found between some genetic polymorphisms and arsenic, lead and cadmium exposure levels may indicate their role in the promotion of obesity and metabolic disorders. These metals may be one of many environmental factors that, in an unfavorable genetic constellation, contribute to higher cardiovascular risk resulting from obesity, diabetes and atherogenic lipid profile. Immunological processes modulated by vitamin D also have their impact on this risk. The relationship between lower cadmium levels and polymorphism of one of the vitamin D receptors proves that this polymorphism is beneficial in reducing cardiovascular risk in persons occupationally exposed to cadmium.

## 5. Conclusions

(1) Single nucleotide polymorphisms within genes coding for proteins involved in development of metabolic syndrome may be of prognostic value for persons directly exposed to lead, cadmium and arsenic.
(2) In the group occupationally exposed to arsenic, cadmium and lead, certain associations between polymorphisms *rs806381*, *rs1049353* and *rs12720071* of gene CNR1 and polymorphism *rs7799039* of gen LEP and arsenic concentration in urine were acquired:
  - Allele G in locus *rs806381* CNR1 and locus *rs7799039* LEP can be responsible for higher arsenic concentrations;
  - Allele A in locus *rs1049353* and *rs12720071* CNR1 can be responsible for lower arsenic concentrations.
(3) Cadmium concentration in blood in people occupationally exposed can be determined by polymorphism of *rs10735810* VDR FokI gene:
  - Allele T in locus *rs10735810* VDR FokI gene can be responsible for lower cadmium concentration.
(4) In people occupationally exposed to arsenic, cadmium and lead, there are certain interactions between polymorphisms *rs1049353* gene CNR1 and *rs9939609* gene FTO and markers of lead exposure (lead and zinc protoporphyrin in blood):
  - Allele A in locus *rs1049353* CNR1 gene can be responsible for lower lead and ZnPP concentrations;
  - Allele A in locus *rs9939609* FTO gene can be responsible for higher ZnPP concentration.
(5) Polymorphism *rs17782813* MC4R gene, as the only one in our study, did not affect concentrations of selected markers amongst workers occupationally exposed to lead, cadmium and arsenic.

## Figures and Tables

**Table 1:** Clinical characteristics of the study population.

	X	Me	SD	Min	Max
Age (years)	49.04	50.00	11.08	26.00	67.00
Height (cm)	174.24	175.50	7.64	156.00	190.00
Weight (kg)	87.39	86.00	15.93	53.00	127.00
BMI (kg/m^2^)	28.68	27.91	4.29	19.00	40.90
Waist circumference (cm)	100.44	100.00	12.55	72.00	125.00
Pack-years	465.41	340.00	386.40	60.00	1500.00
	n		%		
Number	85		100.0		
Gender Male Female	67 18		78.8 21.2		
Weight Normal Overweight Obese	18 33 33		21.2 38.8 38.8		
Smokers	37		43.5		
Hypertension	34		40.0		
Diabetes	10		11.8		

Max-maximal value; Me-median value; Min-minimal value; SD-standard deviation; X-arithmetic mean.

**Table 2:** Conventional lab tests and calcium-phosphate balance in the study population.

	X	Me	SD	Min	Max
WBC (K/µL)	7.25	6.85	1.83	3.83	13.37
RBC (M/µL)	5.04	5.05	0.36	4.28	5.85
Hemoglobin (g/dL)	15.21	15.30	1.01	12.10	17.00
Hematocrit (%)	44.58	44.70	2.70	37.90	50.00
Platelets (K/µL)	249.93	246.00	52.44	129.00	406.00
Glucose (mg/dL)	96.70	93.00	20.80	68.00	181.00
HbA1C (%)	5.71	5.40	1.21	4.60	13.60
Total cholesterol (mg/dL)	234.24	231.00	49.34	102.00	405.00
HDL cholesterol (mg/dL)	51.02	49.00	11.08	27.00	86.00
LDL cholesterol (mg/dL)	138.00	134.00	41.43	27.00	311.00
Triglycerides (mg/dL)	236.58	196.00	155.31	46.00	824.00
Calcium (mg/dL)	9.69	9.70	0.33	9.10	10.60
Phosphorus (mg/dL)	3.43	3.30	0.66	2.20	5.60
25-OH-D_3_ (µg/L)	20.81	20.49	7.69	5.21	45.00
Parathormone (ng/L)	46.68	43.30	20.02	15.50	108.60

**Table 3:** Basic toxicological parameters in the study population.

	X	Me	SD	Min	Max
Exposure period (years)	17.64	13.00	13.33	0.25	46.00
As-U (μg/L)	11.74	9.93	9.37	0.27	46.15
Cd-B (μg/L)	0.84	0.55	0.80	0.22	4.61
Pb-B (μg/L)	199.23	193.80	117.02	22.20	520.90
ZnPP (μg/dL)	47.94	35.00	30.64	21.00	160.00
	***n***		**%**		
Studied population	85		100.0		
As-U >acceptable biological concentration (>35 μg/L)	3		3.5		
Cd-B >acceptable biological concentration (>5 μg/L)	0		0.0		
Pb-B >acceptable biological concentration (>500 μg/L)	1		1.2		
ZnPP>acceptable biological concentration (>70 μg/dL)	14		16.5		

**Table 4:** Selected polymorphisms of genes CNR1, MC4R, LEP, FTO and VDR FokI in the study population.

SNP	Genotype	*n*	%	Allele	*n*	%
*rs806381* gene CNR1	homozygote AA heterozygote AG homozygote GG	27 43 15	31.8 50.6 17.6	allele A allele G	70 58	82.4 68.2
*rs806368* gene CNR1	homozygote CC heterozygote CT homozygote TT	7 23 55	8.2 27.1 64.7	allele C allele T	30 78	35.3 91.8
*rs1049353* gene CNR1	homozygote AA heterozygote AG homozygote GG	5 34 46	5.9 40.0 54.1	allele A allele G	39 80	45.8 94.1
*rs12720071* gene CNR1	homozygote AA heterozygote AG homozygote GG	69 15 1	81.2 17.6 1.2	allele A allele G	84 16	98.8 18.8
*rs17782313* gene MC4R	homozygote CC heterozygote CT homozygote TT	5 27 53	5.9 31.8 62.4	allele C allele T	32 80	37.6 94.1
*rs7799039* gene LEP	homozygote AA heterozygote AG homozygote GG	14 40 30	16.5 47.1 35.3	allele A allele G	54 70	63.5 82.3
*rs9939609* gene FTO	homozygote AA heterozygote AT homozygote TT	23 43 19	27.1 50.6 22.4	allele A allele T	66 62	77.6 72.4
*rs10735810* gene VDR FokI	homozygote CC heterozygote CT homozygote TT	25 41 19	29.4 48.2 22.4	allele C allele T	66 60	77.6 70.6

**Table 5:** Total arsenic concentration in subgroups divided according to selected polymorphisms of genes CNR1, MC4R, LEP, FTO and VDR FokI.

SNP	Genotype	As-U (μg/L)	Allele	As-U (μg/L)
*rs806381* gene CNR1	homozygote AA heterozygote AG homozygote GG	8.58 ± 5.38 12.81 ± 10.00 14.56 ± 12.11	allele A allele G	11.18 ± 8.72 13.24 ± 10.47
	AA vs. AG: *p* = 0.044 AA vs. GG: *p* = 0.041		G (GG or AG) vs. non-G (AA): *p* = 0.032	
*rs806368* gene CNR1	homozygote CC heterozygote CT homozygote TT	11.43 ± 9.61 12.39 ± 11.85 11.51 ± 8.31	allele C allele T	12.19 ± 11.27 11.77 ± 9.42
	ns		ns	
*rs1049353* gene CNR1	homozygote AA heterozygote AG homozygote GG	8.99 ± 4.68 9.56 ± 6.96 13.70 ± 10.91	allele A allele G	9.48 ± 6.66 11.92 ± 9.58
	ns		A (AA or AG) vs. non-A (GG): *p* = 0.039	
*rs12720071* gene CNR1	homozygote AA heterozygote AG homozygote GG	11.04 ± 8.44 13.72 ± 12.26 29.58 ± 0.00	allele A allele G	11.53 ± 9.22 14.72 ± 12.49
	AA vs. GG: *p* = 0.034 AG vs. GG: *p* = 0.039		A (AA or AG) vs. non-A (GG): *p* = 0.045	
*rs17782313* gene MC4R	homozygote CC heterozygote CT homozygote TT	14.06 ± 5.78 10.25 ± 8.36 12.30 ± 10.13	allele C allele T	10.84 ± 8.06 11.60 ± 9.56
	ns		ns	
*rs7799039* gene LEP	homozygote AA heterozygote AG homozygote GG	7.76 ± 5.27 12.11 ± 9.98 12.60 ± 9.52	allele A allele G	10.98 ± 9.15 12.31 ± 9.72
	ns		G (GG or AG) vs. non-G (AA): *p* = 0.043	
*rs9939609* gene FTO	homozygote AA heterozygote AT homozygote TT	10.13 ± 7.14 13.10 ± 10.87 10.54 ± 7.79	allele A allele T	12.09 ± 9.81 12.31 ± 10.04
	ns		ns	
*rs10735810* gene VDR FokI	homozygote CC heterozygote CT homozygote TT	13.58 ± 11.47 9.97 ± 7.64 13.21 ± 9.54	allele C allele T	11.34 ± 9.36 10.96 ± 8.32
	ns		ns	

ns-non-significant statistically.

**Table 6:** Cadmium concentration in subgroups divided according to selected polymorphisms of genes CNR1, MC4R, LEP, FTO and VDR FokI.

SNP	Genotype	Cd-B (μg/L)	Allele	Cd-B (μg/L)
*rs806381* gene CNR1	homozygote AA heterozygote AG homozygote GG	0.89 ± 0.98 0.85 ± 0.75 0.75 ± 0.60	allele A allele G	0.86 ± 0.84 0.82 ± 0.71
	ns		ns	
*rs806368* gene CNR1	homozygote CC heterozygote CT homozygote TT	0.66 ± 0.35 0.89 ± 0.94 0.85 ± 0.79	allele C allele T	0.84 ± 0.84 0.86 ± 0.83
	ns		ns	
*rs1049353* gene CNR1	homozygote AA heterozygote AG homozygote GG	1.08 ± 1.05 0.74 ± 0.61 0.90 ± 0.90	allele A allele G	0.78 ± 0.67 0.83 ± 0.79
	ns		ns	
*rs12720071* gene CNR1	homozygote AA heterozygote AG homozygote GG	0.83 ± 0.74 0.95 ± 1.07 0.35 ± 0.00	allele A allele G	0.85 ± 0.80 0.92 ± 1.04
	ns		ns	
*rs17782313* gene MC4R	homozygote CC heterozygote CT homozygote TT	1.08 ± 1.05 0.73 ± 0.61 0.88 ± 0.87	allele C allele T	0.79 ± 0.69 0.83 ± 0.79
	ns		ns	
*rs7799039* gene LEP	homozygote AA heterozygote AG homozygote GG	0.95 ± 0.75 0.86 ± 0.89 0.77 ± 0.73	allele A allele G	0.89 ± 0.85 0.82 ± 0.82
	ns		ns	
*rs9939609* gene FTO	homozygote AA heterozygote AT homozygote TT	0.74 ± 0.64 0.81 ± 0.78 1.04 ± 1.01	allele A allele T	0.79 ± 0.73 0.88 ± 0.85
	ns		ns	
*rs10735810* gene VDR FokI	homozygote CC heterozygote CT homozygote TT	1.12 ± 1.17 0.68 ± 0.46 0.82 ± 0.74	allele C allele T	0.85 ± 0.82 0.73 ± 0.56
	ns		T (TT or CT) vs. non-T (CC): *p* = 0.041	

**Table 7:** Lead concentration in subgroups divided according to selected polymorphisms of genes CNR1, MC4R, LEP, FTO and VDR FokI.

SNP	Genotype	Pb-B (μg/L)	Allele	Pb-B (μg/L)
*rs806381* gene CNR1	homozygote AA heterozygote AG homozygote GG	180.50 ± 97.72 202.85 ± 137.05 222.58 ± 82.32	allele A allele G	194.23 ± 123.09 207.95 ± 124.82
	ns		ns	
*rs806368* gene CNR1	homozygote CC heterozygote CT homozygote TT	207.09 ± 50.81 172.01 ± 111.61 209.61 ± 124.52	allele C allele T	180.20 ± 101.05 198.53 ± 121.37
	ns		ns	
*rs1049353* gene CNR1	homozygote AA heterozygote AG homozygote GG	171.58 ± 117.21 160.00 ± 107.64 231.23 ± 116.39	allele A allele G	161.48 ± 107.35 200.96 ± 117.53
	AG vs. GG: *p* = 0.006 AA vs. GG: *p* = 0.045		A (AA or AG) vs. non-A (GG): *p* = 0.005	
*rs12720071* gene CNR1	homozygote AA heterozygote AG homozygote GG	193.85 ± 115.37 224.46 ± 129.17 192.20 ± 0.00	allele A allele G	199.31 ± 117.72 222.44 ± 125.05
	ns		ns	
*rs17782313* gene MC4R	homozygote CC heterozygote CT homozygote TT	244.16 ± 99.42 174.66 ± 116.00 207.51 ± 118.55	allele C allele T	185.52 ± 114.97 196.42 ± 118.00
	ns		ns	
*rs7799039* gene LEP	homozygote AA heterozygote AG homozygote GG	208.89 ± 137.93 193.02 ± 119.09 201.17 ± 108.88	allele A allele G	197.13 ± 123.09 196.51 ± 114.08
	ns		ns	
*rs9939609* gene FTO	homozygote AA heterozygote AT homozygote TT	205.35 ± 107.83 206.36 ± 127.10 175.69 ± 106.02	allele A allele T	206.01 ± 119.89 196.96 ± 121.01
	ns		ns	
*rs10735810* gene VDR FokI	homozygote CC heterozygote CT homozygote TT	224.20 ± 139.97 175.76 ± 107.16 217.04 ± 99.02	allele C allele T	194.11 ± 121.91 188.83 ± 105.60
	ns		ns	

**Table 8:** Zinc protoporphyrins (ZnPP) concentration in subgroups divided according to selected polymorphisms of genes CNR1, MC4R, LEP, FTO and VDR FokI.

SNP	Genotype	ZnPP (μg/dL)	Allele	ZnPP (μg/dL)
*rs806381* gene CNR1	homozygote AA heterozygote AG homozygote GG	42.48 ± 25.15 48.19 ± 29.47 57.07 ± 41.30	allele A allele G	45.99 ± 27.84 50.48 ± 32.78
	ns		ns	
*rs806368* gene CNR1	homozygote CC heterozygote CT homozygote TT	64.86 ± 48.64 41.70 ± 24.01 48.40 ± 30.15	allele C allele T	47.10 ± 32.03 46.42 ± 28.49
	ns		ns	
*rs1049353* gene CNR1	homozygote AA heterozygote AG homozygote GG	56.60 ± 42.48 37.15 ± 18.61 54.98 ± 34.51	allele A allele G	39.64 ± 23.11 47.40 ± 30.03
	AG vs. GG: *p* = 0.009 AA vs. AG: *p* = 0.037		A (AA or AG) vs. non-A (GG): *p*=0.020	
*rs12720071* gene CNR1	homozygote AA heterozygote AG homozygote GG	47.38 ± 30.92 47.47 ± 28.91 94.00 ± 0.00	allele A allele G	47.39 ± 30.40 50.38 ± 30.26
	ns		ns	
*rs17782313* gene MC4R	homozygote CC heterozygote CT homozygote TT	51.20 ± 43.25 44.89 ± 31.59 49.19 ± 29.43	allele C allele T	45.88 ± 32.92 47.74 ± 30.05
	ns		ns	
*rs7799039* gene LEP	homozygote AA heterozygote AG homozygote GG	53.57 ± 32.82 42.75 ± 25.72 51.57 ± 35.59	allele A allele G	46.53 ± 30.42 45.56 ± 27.82
	ns		ns	
*rs9939609* gene FTO	homozygote AA heterozygote AT homozygote TT	52.26 ± 37.44 50.44 ± 29.70 37.05 ± 21.13	allele A allele T	51.08 ± 32.33 46.34 ± 27.89
	ns		A (AA or AT) vs. non-A (TT): *p* = 0.038	
*rs10735810* gene VDR FokI	homozygote CC heterozygote CT homozygote TT	52.96 ± 29.89 42.17 ± 28.05 53.79 ± 35.97	allele C allele T	46.26 ± 29.02 45.85 ± 30.95
	ns		ns	

**Table 9:** Results of estimation for the final model obtained from multivariate regression analysis.

	Independent Variable	Regression Coefficient	Standard error of Regression Coefficient	*p*-Value	*p* Value of the Model	Standard Error of the Model
model for As-U (μg/L)	allele G in *rs806381* gene CNR1	1.330	0.666	0.041	0.043	4.877
	allele A in *rs1049353* gene CNR1	−4.274	2.095	0.037		
	allele A in *rs12720071* gene CNR1	−18.415	9.136	0.037		
	allele G in *rs7799039* gene LEP	2.291	1.072	0.045		
	BMI (kg/m^2^)	0.424	0.173	0.025		
	age (years)	0.160	0.070	0.015		
	diabetes	5.324	2.269	0.026		
model for Cd-B (μg/L)	allele T in *rs10735810* gene VDR FokI	−0.439	0.187	0.022	0.006	0.709
	smoking	0.814	0.184	0.001		
	HDL cholesterol (mg/dL)	−0.023	0.011	0.045		
	BMI (kg/m2)	0.014	0.002	0.046		
model for Pb-B (μg/L)	allele A in *rs1049353* gene CNR1	−77.411	26.320	0.004	0.014	112.131
	hypertension	62.804	39.182	0.014		
	diabetes	82.478	58.742	0.016		
	phosphorus (mg/dL)	−44.553	21.391	0.041		
